# Autism spectrum disorder research: knowledge mapping of progress and focus between 2011 and 2022

**DOI:** 10.3389/fpsyt.2023.1096769

**Published:** 2023-04-25

**Authors:** Miaomiao Jiang, Tianlan Lu, Kang Yang, Xianjing Li, Liyang Zhao, Dai Zhang, Jun Li, Lifang Wang

**Affiliations:** ^1^National Clinical Research Center for Mental Disorders (Peking University Sixth Hospital), NHC Key Laboratory of Mental Health (Peking University), Peking University Sixth Hospital, Peking University Institute of Mental Health, Beijing, China; ^2^Translational Medicine Center of Chinese Institute for Brain Research, Beijing, China; ^3^Guangdong Key Laboratory of Mental Health and Cognitive Science, Institute for Brain Research and Rehabilitation (IBRR), South China Normal University, Guangzhou, China

**Keywords:** autism spectrum disorder, bibliometric study, CiteSpace, VOSviewer, research frontiers

## Abstract

**Background:**

In recent years, a large number of studies have focused on autism spectrum disorder (ASD). The present study used bibliometric analysis to describe the state of ASD research over the past decade and identify its trends and research fronts.

**Methods:**

Studies on ASD published from 2011 to 2022 were obtained from the Web of Science Core Collection (WoSCC). Bibliometrix, CiteSpace, and VOSviewer were used for bibliometric analysis.

**Results:**

A total of 57,108 studies were included in the systematic search, and articles were published in more than 6,000 journals. The number of publications increased by 181.7% (2,623 in 2011 and 7,390 in 2021). The articles in the field of genetics are widely cited in immunology, clinical research, and psychological research. Keywords co-occurrence analysis revealed that “causative mechanisms,” “clinical features,” and “intervention features” were the three main clusters of ASD research. Over the past decade, genetic variants associated with ASD have gained increasing attention, and immune dysbiosis and gut microbiota are the new development frontiers after 2015.

**Conclusion:**

This study uses a bibliometric approach to visualize and quantitatively describe autism research over the last decade. Neuroscience, genetics, brain imaging studies, and gut microbiome studies improve our understanding of autism. In addition, the microbe-gut-brain axis may be an exciting research direction for ASD in the future. Therefore, through visual analysis of autism literature, this paper shows the development process, research hotspots, and cutting-edge trends in this field to provide theoretical reference for the development of autism in the future.

## Introduction

Autism spectrum disorder (ASD) refers to a group of early-onset, lifelong, heterogeneous neurodevelopmental conditions with complex mechanisms of emergence (1). The prevalence of ASD has increased from 1 in 69 by 2012 to 1 in 44 by 2018, as reported by the Centers for Disease Control and Prevention for 2012–2018 (2, 3). Recent research estimates the male-to-female ratio is closer to 2:1 or 3:1, indicating a higher diagnostic prevalence of autism in males compared to females (4–6). Some studies have shown a high heritability of 80–93% in ASD and reported hundreds of risk gene loci (7).

Specific autistic characteristics usually appear before the age of 3 years, and some children on the spectrum may have limited nonverbal and verbal communication by the age of 18–24 months (8, 9). The diagnosis of ASD is based on the core features of social communication impairment and unusual and repetitive sensory-motor behavior (10). Some autistic individuals can be definitively diagnosed with autism as early as 2–3 years of age and the mean age of diagnosis for autistic children is still 4–5 years (1, 11). It is important to stress that more adults are getting assessed for possible autism (5). As autism is increasingly diagnosed, multidisciplinary involvement can help have a positive impact on the well-being and quality of life for both children and adults on the spectrum (12). Several mental diseases also affect autistic individuals, increasing the diagnosis complexity (13).

Over the past decade, researchers have struggled to explain the neurological etiology, and great progress has been made in the genetics, epigenetics, neuropathology, and neuroimaging of ASD (9). However, there is a lack of systematic review of field research and discussion of future research hotspots. Bibliometrics (14) belongs to interdisciplinary research, which has been widely used in science by analyzing highly cited papers, field keyword clustering, and the internal cooperation links of countries, thus providing a comprehensive interpretation of the development process of autism research field (15).

In some of the previous bibliometrics studies on ASD, a single software was used to focus on a specific field or research aspect of the autism (16–18), and the trend in the past decade has not yet been displayed. The present study comprehensively combines Bibliometrix package, CiteSpace, and VOSviewer to (1) dynamically assess quantitative indicators of ASD research publications and use different index indicators to measure the quality of research; (2) further identify the most contributing countries, institutions, journals, and authors; (3) analyze the citation network architecture; (4) determine the top 100 most cited papers; (5) conduct keyword analysis. Subsequently, bibliometrics was used to understand the current hotspots and trends in the field of ASD research for further in-depth investigation.

## Materials and methods

### Data collection and search strategies

**Figure 1:**
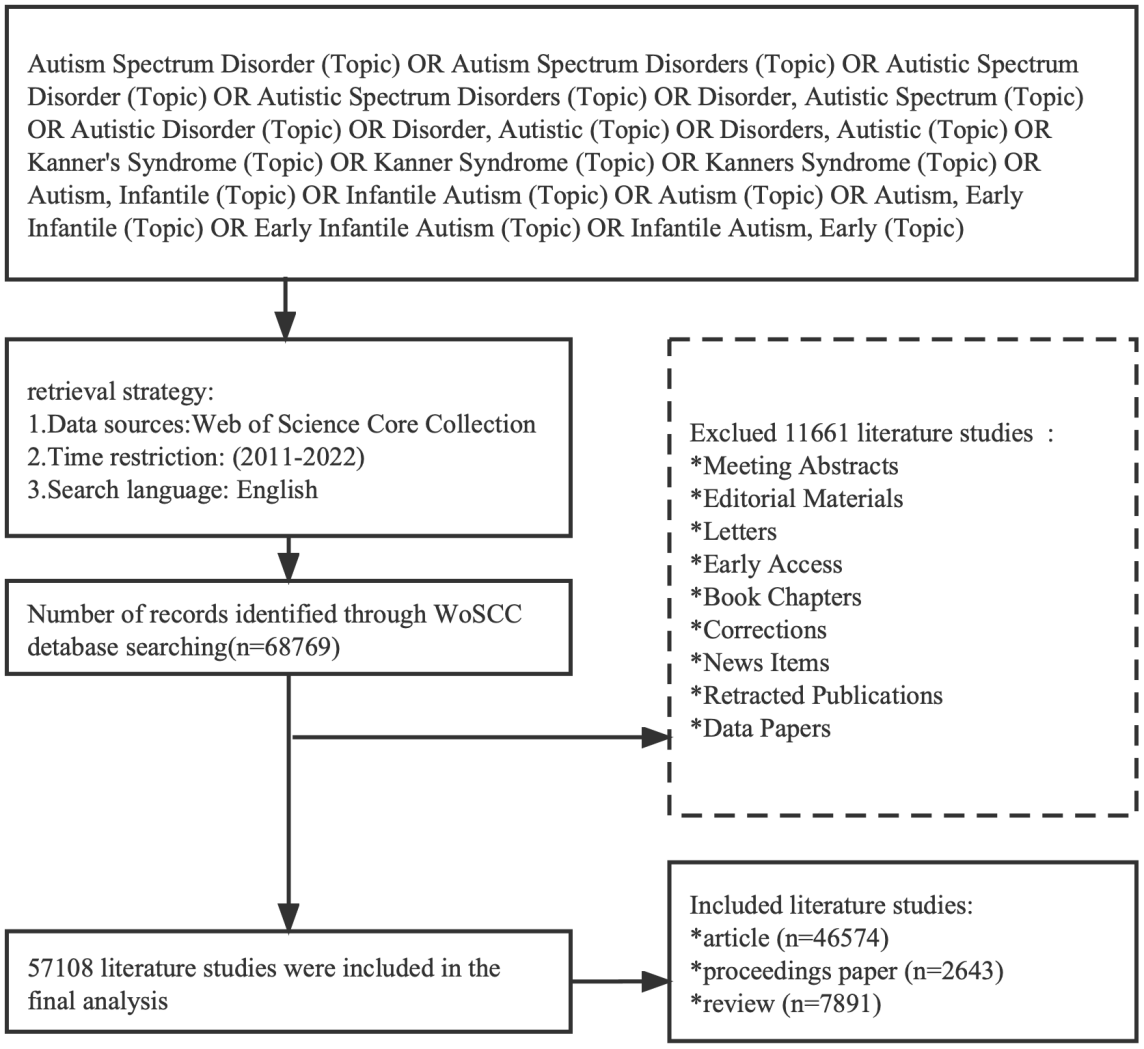
Flowchart of the screening process.

We comprehensively searched the Web of Science Core Collection (WoSCC) database from 2011 to 2022. WoSCC is a daily updated database covering an abstract index of multidisciplinary literature that exports complete citation data, maintained by Thomson Reuters (New York, NY, USA) (19). The articles’ data were independently searched by two researchers on May 29, 2022, to avoid bias caused by database updates. The scientometric retrieval process is illustrated in Figure 1. A total of 68,769 original articles in English language were retrieved, excluding 11,661 irrelevant articles, such as meeting abstracts, editorial materials, corrections, and letters. A total of 57,108 documents were exported, and the retrieved documents would be exported in the form of all records and references.

### Grey prediction model

Grey models (GM) are used to construct differential prediction models with limited and incomplete data (20). The GM (1,1) model, with high accuracy and convenient calculations, is extensively utilized in the energy and medical industries (21). We used the standard GM (1,1) model to forecast the annual publication volume over the next 5 years. The operation of GM (1,1) model was done by using Python software.

### Bibliometric analysis and visualization

The records of the retrieved publications were exported to Bibliometrix, CiteSpace, and VOSviewer for further bibliometric analysis.

Bibliometrix package (running on R4.0.3) was utilized to capture and extract the bibliographic information on selected publications, including topic, author, keywords, and country distribution (22). The productivity of authors/journals in the field was measured by the number of publications (Np) and assessing metrics, such as the number of citations, publication h-index value, and m-index value. The h-index is used to quantify the scientific output and measure the citation impact, and two people with similar h-index may have a similar impact in the scientific field, even if the total number of papers or total citations are different (23). The m-index can be used to compare the influence of scholars with different academic career years. The number of citations of a document is a measure of its scientific impact to a certain extent (24). Bibliometrix package was also used to screen the top 100 articles and explore research trends and hotspots.

VOSviewer is a free computer program to visualize bibliometric maps (25). The keyword co-occurrence network was constructed using VOSviewer. CiteSpace is based on the Java environment and uses methods, such as co-occurrence analysis and cluster analysis, for the visualization of scientific literature research data in specific disciplines. The visual knowledge maps were constructed using the procedural steps of CiteSpace (26), including time slicing, threshold, pruning, merging, and mapping; then, the contribution of countries and institutions of ASD over the past decade was assessed based on centrality scores. The co-citation network and dual-map of references were constructed by CiteSpace. A dual-map (27) overlay is a bipartite overlay analysis method by CiteSspace, which uses the distribution map cited journals in the WoS database as the base map, and the map generated by the cited literature data as the overlay map.

## Results

### Annual publications

**Figure 2:**
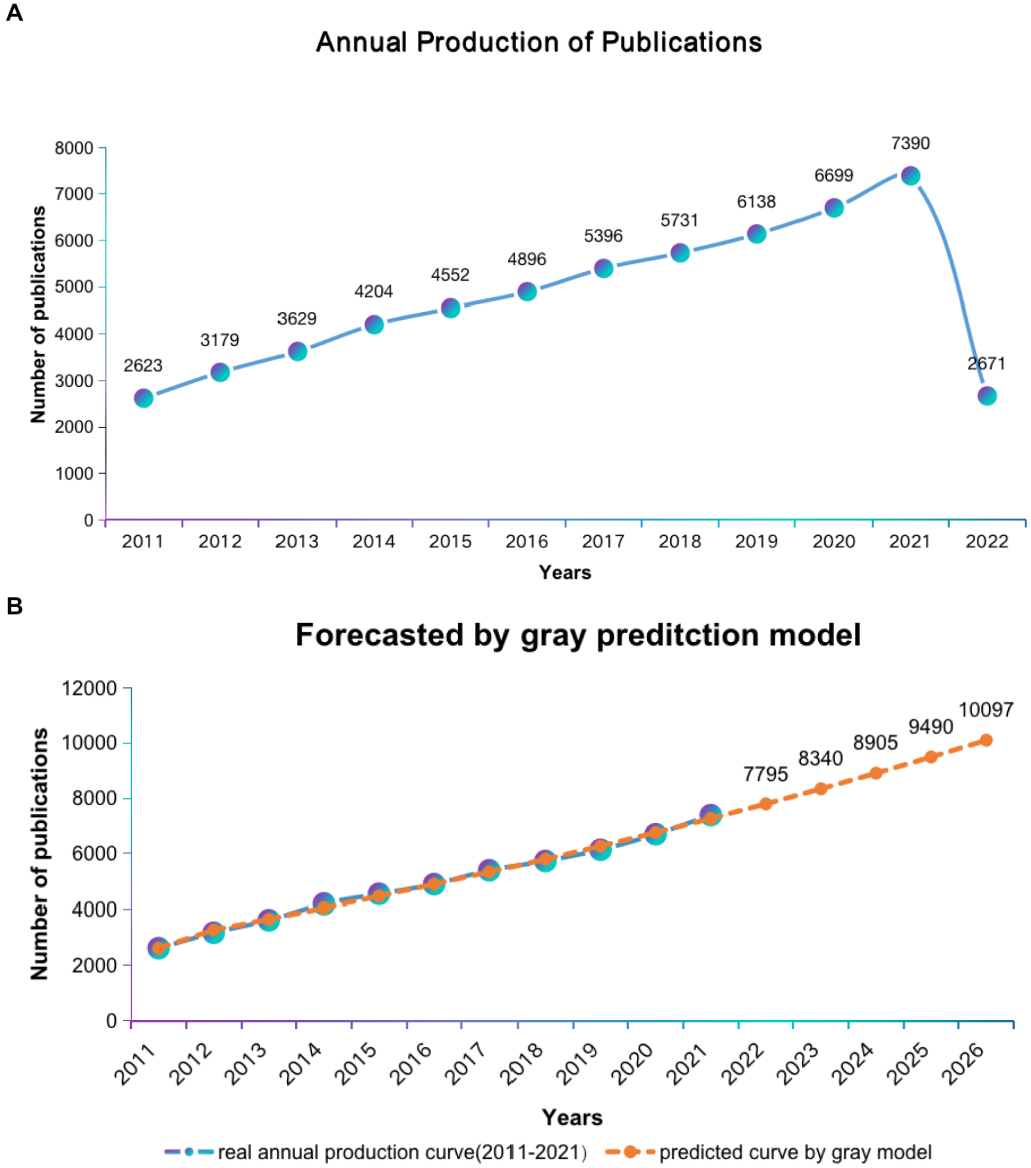
Global trends in publications of ASD research. **(A)** Single-year publication output over the past decade. **(B)** Model forecast curves for publication growth trends.

A total of 57,108 articles were included in this study, consisting of 46,574 articles, 2,643 conference papers, and 7,891 reviews. From 2011 to 2022, the number of publications maintained a steady growth rate (Figure 2A), and the grey prediction model predicted the trend of increasing publication volume in the next 5 years (Figure 2B). The main information for all publications is shown in Supplementary Table S1.

### Distribution of countries and institutions

**Figure 3:**
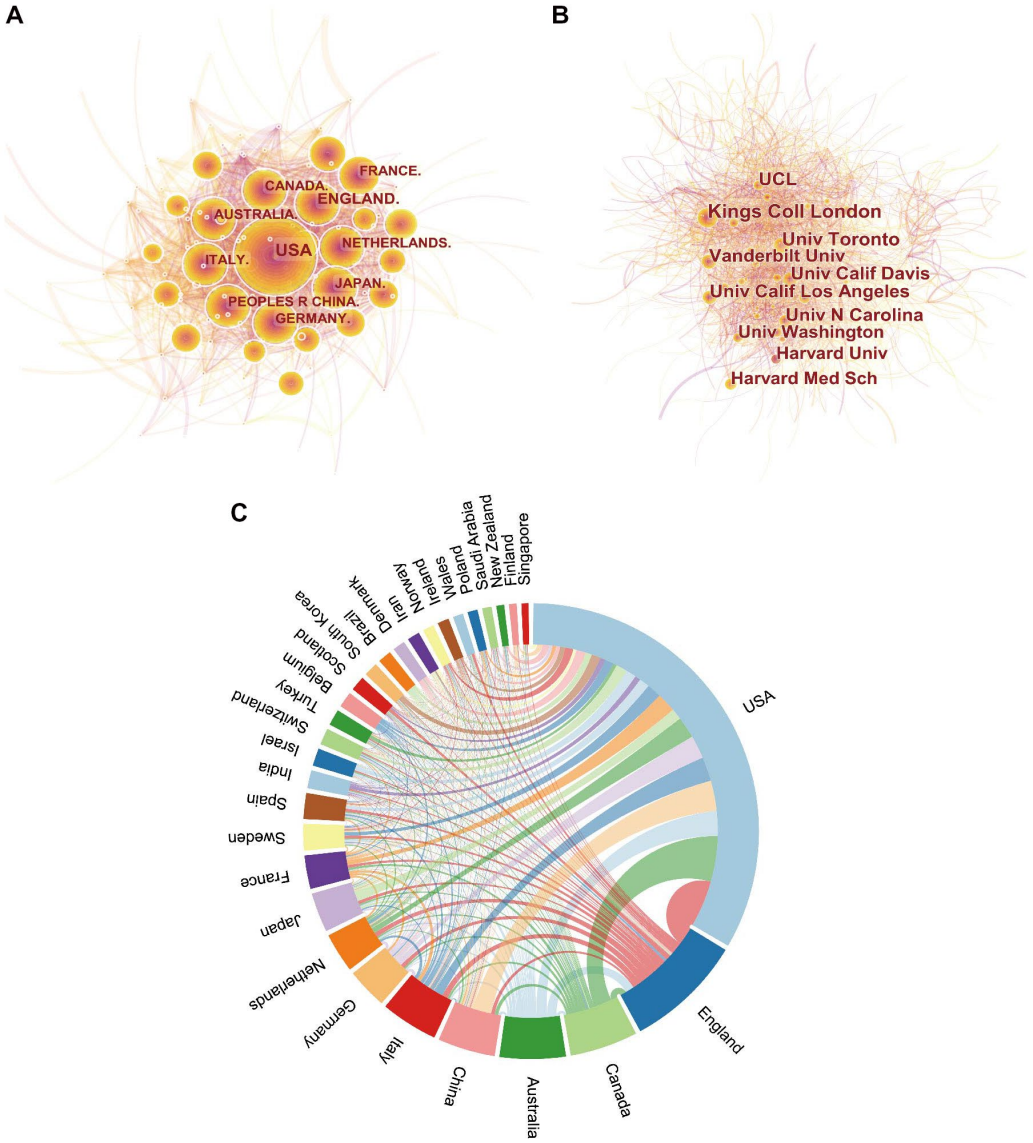
The distribution of countries and institutions. Map of countries **(A)** and institutions **(B)** contributed to publications related to ASD research. **(C)** Network diagram showing international collaborations involved in ASD research. The nodes represent the countries and institutions; the color depth and size of the circle are positively correlated to the number of posts. The thickness of the curved connecting lines represents the strength of collaboration in the countries and institutions.

Autism-related research has been conducted by researchers from a variety of countries and institutions, and articles in this field have been cited 1,231,588 times (Tables 1, 2). CiteSpace visualizes collaborative networks between institutions and countries (Figures 3A,B). As shown in the international collaborations network of autism research (Figure 3C), the USA and UK are the leading countries working closely with other countries.

**Table 1:** Publications in top 10 most productive countries.

Countries	Ranking based on output	Output^a^ *n* (%)	SCP^b^	MCP^c^	Ranking based on citations	Total citation^d^	Average article citation
USA	1	22,615 (39.60)	19,373	3,242	1	616,323	27.25
UK	2	4,961 (8.69)	3,440	1,521	2	123,685	24.93
China	3	3,211 (5.63)	2,357	854	6	40,561	12.63
Australia	4	2,659 (4.65)	1870	789	4	52,335	19.68
Canada	5	2,582 (4.52)	1794	788	3	60,919	23.59
Italy	6	2,317 (4.06)	1,656	661	5	42,136	18.19
Japan	7	1883 (3.29)	1,572	311	9	24,927	13.24
Netherlands	8	1,362 (2.38)	857	505	7	35,425	26.01
Germany	9	1,246 (2.18)	718	528	8	33,395	26.8
France	10	1,126 (1.97)	689	437	10	24,579	21.83

a *N* = 57,108.

b Articles in which all authors have the same country affiliation are called single country publications (SCP) and are considered to represent intra-country (within) collaboration.

c Articles with authors having different country affiliations are called multiple country publications (MCP) and considered to represent the international collaboration of that country.

d *N* = 1,231,588.

**Table 2:** Publications in top 10 most productive Institutions.

Institutions	Country	Counts
Kings College London	UK	1,214
University of Toronto	Canada	1,022
Vanderbilt University	USA	978
University of California, Davis	USA	938
University of California, Los Angeles	USA	910
University of North Carolina	USA	863
University College London	UK	836
University of Washington	USA	794
Harvard University	USA	776
Harvard Medical School	USA	775

### Analysis of journals

**Figure 4:**
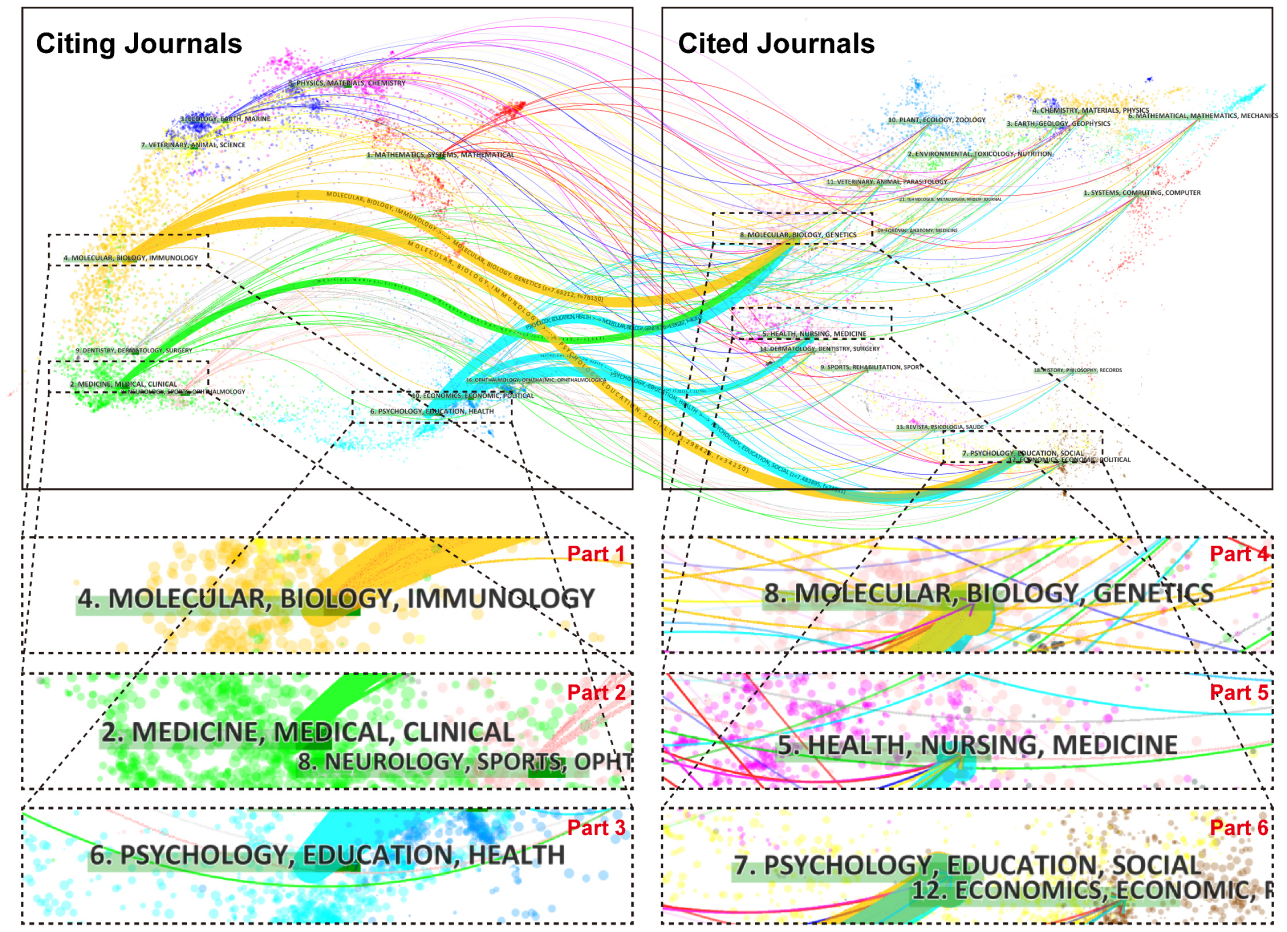
A dual-map overlay of journals that published work related to ASD. A presentation of citation paths at a disciplinary level on a dual-map overlay. The width of the paths is proportional to the z-score-scale citation frequency. The labels on the map represent the research subjects covered by the journals, and the wavy curve connects the citing articles on the left side of the map and the cited articles on the right side of the map.

The h-index combines productivity and impact; typically, a high h-index means a high recognition. As presented in Table 3, the Journal of Autism and Developmental Disorders, PLOS One, and Molecular Psychiatry were among the top three of the 20 journals with the highest h-index. The Journal of Autism and Developmental Disorders has the highest number of articles (3478) and cited number of publications (90308). Among the top 20, four journals with impact factors >10 include Molecular Psychiatry (IF: 13.437), Biological Psychiatry (IF: 12.810), Proceedings of the National Academy of Sciences of the United States of America (IF: 12.779), Journal of the American Academy of Child and Adolescent Psychiatry (IF: 13.113), which have been cited more than 10,000 times. In addition, 75% of journals belong to Q1 (Table 3). The cited journals provided the knowledge base of the citing journals. The yellow paths illustrate that studies published in “molecular, biology, immunology” journals tended to cite journals primarily in the domains of “molecular, biology, genetics,” and “psychology, education, social.” The paths colored with grass-green paths illustrate that studies published in “medicine, medical, clinical” journals tended to cite journals primarily in the domains of “molecular, biology, and genetics.” The pale blue paths showcase that research published in “psychology, education, health” journals preferred to quote journals mostly in the domains of “molecular, biology, genetics,” “health, nursing, medicine,” and “psychology, education, social (Figure 4).”

**Table 3:** Top 20 journals ranked by h_index.

Rank	Name	h_index	Count	TC	IF (2022)	JCR (2022)
1	Journal of Autism and Developmental Disorders	110	3,478	90,308	4.345	Q2
2	PloS One	75	856	27,049	3.752	Q2
3	Molecular Psychiatry	74	292	18,125	13.437	Q1
4	Autism	73	1,130	27,510	6.684	Q1
5	Pediatrics	71	227	17,360	9.703	Q1
6	Biological Psychiatry	70	222	13,457	12.810	Q1
7	Proceedings of the National Academy of Sciences of the United States of America	70	199	12,960	12.779	Q1
8	Research in Autism Spectrum Disorders	68	1,289	26,452	3.293	Q3
9	Journal of Child Psychology and Psychiatry	67	281	14,921	8.265	Q1
10	Autism Research	64	1,154	24,293	4.633	Q1
11	Molecular Autism	61	577	17,470	6.476	Q1
12	Neuroscience and Biobehavioral Reviews	60	220	12,396	9.052	Q1
13	Translational Psychiatry	59	344	11,574	7.989	Q1
14	Journal of the American Academy of Child and Adolescent Psychiatry	57	184	12,313	13.113	Q1
15	Research in Developmental Disabilities	56	711	14,422	3.000	Q1
16	Journal of Neuroscience	54	220	10,231	6.709	Q1
17	Frontiers in Human Neuroscience	47	241	7,842	3.473	Q3
18	Human Molecular Genetics	47	163	6,846	5.121	Q1
19	Neuroimage	47	156	7,508	7.400	Q1
20	Journal of Neurodevelopmental Disorders	45	259	6,856	4.074	Q2

TC: total citation; IF: impact factor.

### Analysis of authors

The top 10 most effective authors who have contributed to autism research are listed in Table 4. The g-index and m-index are derivatives of the h-index, and if scientists publish at least 10 articles, of which 7 papers have been cited cumulatively 51 (>49), the g-index is 7; the m-index is related to the academic age of the scientists. The large g-index, h-index, and m-index indicate a great influence on the scholar’s academic influence and high academic achievement. Professor Catherine Lord from the USA is ranked first and has made outstanding contributions to autism research over the past 10 years. In terms of the number of publications, Simon Baron-Cohen was the most productive author (*n* = 278), followed by Tony Charman (*n* = 212) and Christopher Gillberg (*n* = 206). In terms of citations in this field, Daniel H. Geschwind was ranked first (18,127 citations), followed by Catherine Lord (14,830 citations) and Joseph D. Buxbaum (14,528 citations).

**Table 4:** Top 10 most effective authors contributing to autism research.

Author	Country	h_index	g_index	m_index	TC	NP
Catherine Lord	USA	64	121	5.333	14,830	146
Simon Baron-Cohen	UK	60	109	5	14,432	278
Daniel H. Geschwind	USA	58	103	4.833	18,127	103
Lonnie Zwaigenbaum	Canada	57	106	4.75	12,246	193
Tony Charman	UK	55	89	4.583	9,514	212
Stephen W. Scherer	USA	51	115	4.25	13,444	136
Christopher Gillberg	Sweden	48	83	4	8,193	206
Joseph D. Buxbaum	USA	48	120	4	14,528	123
Paul Lichtenstein	Sweden	47	93	3.917	8,898	132
Evan E. Eichler	USA	47	96	3.917	13,393	96

TC: total citation; NP: number of papers.

### Analysis of reference

**Figure 5:**
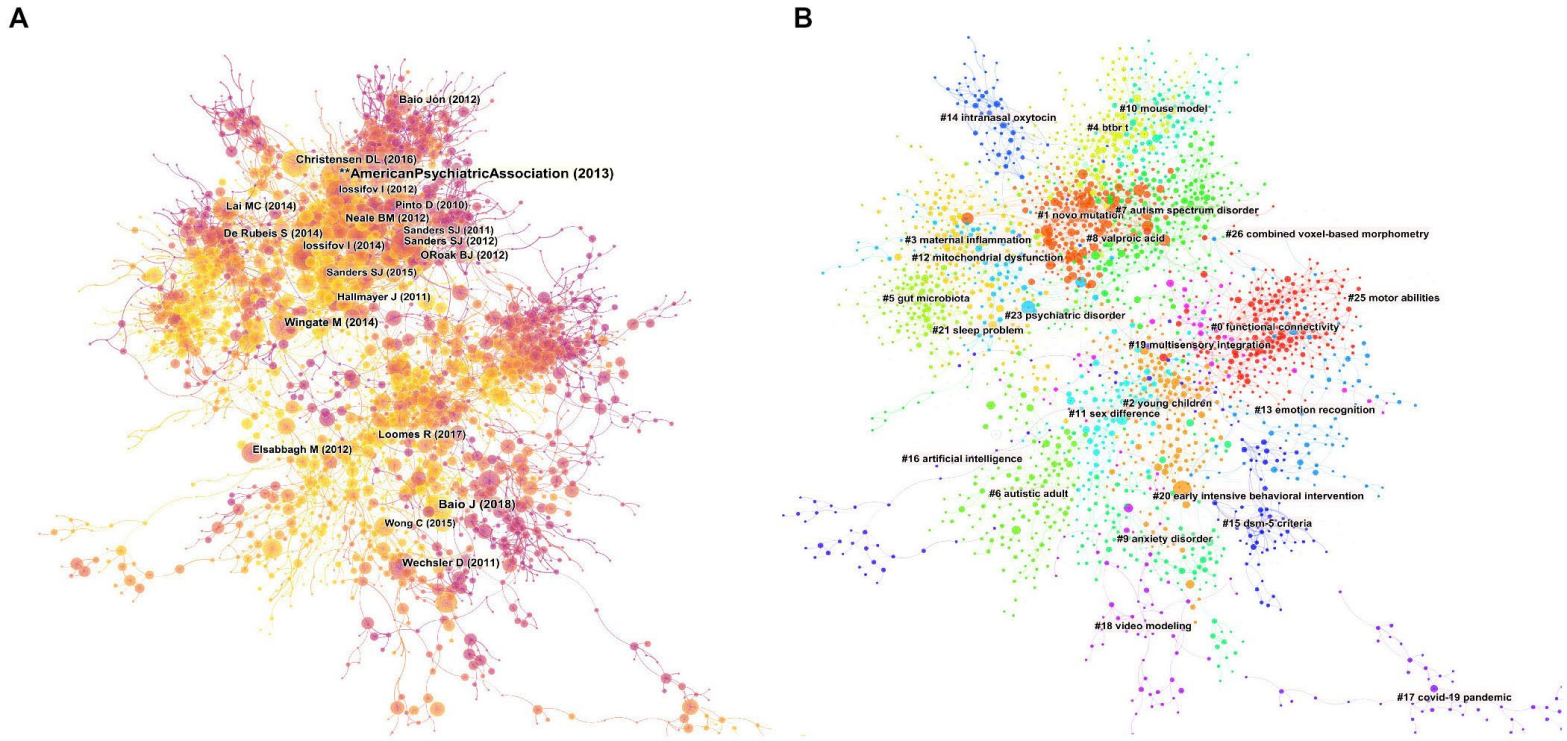
Mapping on co-cited references. **(A)** A network map showing the co-cited references. **(B)** Co-cited clusters with cluster labels.

The co-citation analysis network of 1,056,125 references (Figure 5A) showed that two articles appear simultaneously in the bibliography of the third cited document. The top 20 co-cited references (over the past decade) summarized in ASD studies are listed in Supplementary Table S2. Most of this highly cited literature focuses on the genetic field, discovering genetic risk loci and associated mutations, constructing mutation networks highly associated with autism, and identifying genes associated with autism synaptic destruction. Some studies indicated that *de novo* mutations in ASD might partially explain the etiology. Multiple studies have revealed genetic variants associated with ASD, such as rare copy number variants (CNVs), *de novo* likely gene-disrupting (LGD) mutations, missense or nonsense *de novo* variants, and *de novo* duplications. In the cluster network graph, different colors represent varied clusters, and each node represents a cited paper, displaying the distribution of topics in the field (Figure 5B). The network is divided into 25 co-citation clusters (Figure 5B), primarily related to the diagnosis, etiology, and intervention of autism. The etiological studies include five clusters, *de novo* mutation, inflammation, gut microbiota, mitochondrial dysfunction, and mouse model. Intervention literature focuses on early intensive behavioral intervention, intranasal oxytocin, video modeling, and multisensory integration. The diagnostic aspects of ASD include neuroimaging functional connectivity and Diagnostic and Statistical Manual of Mental Disorders (DSM-5). In addition, some of the references focus on gender/sex differences and sleep problems. Coronavirus disease 2019 (COVID-19) is a new cluster for autism research.

### Co-occurrence analysis of keywords

**Figure 6:**
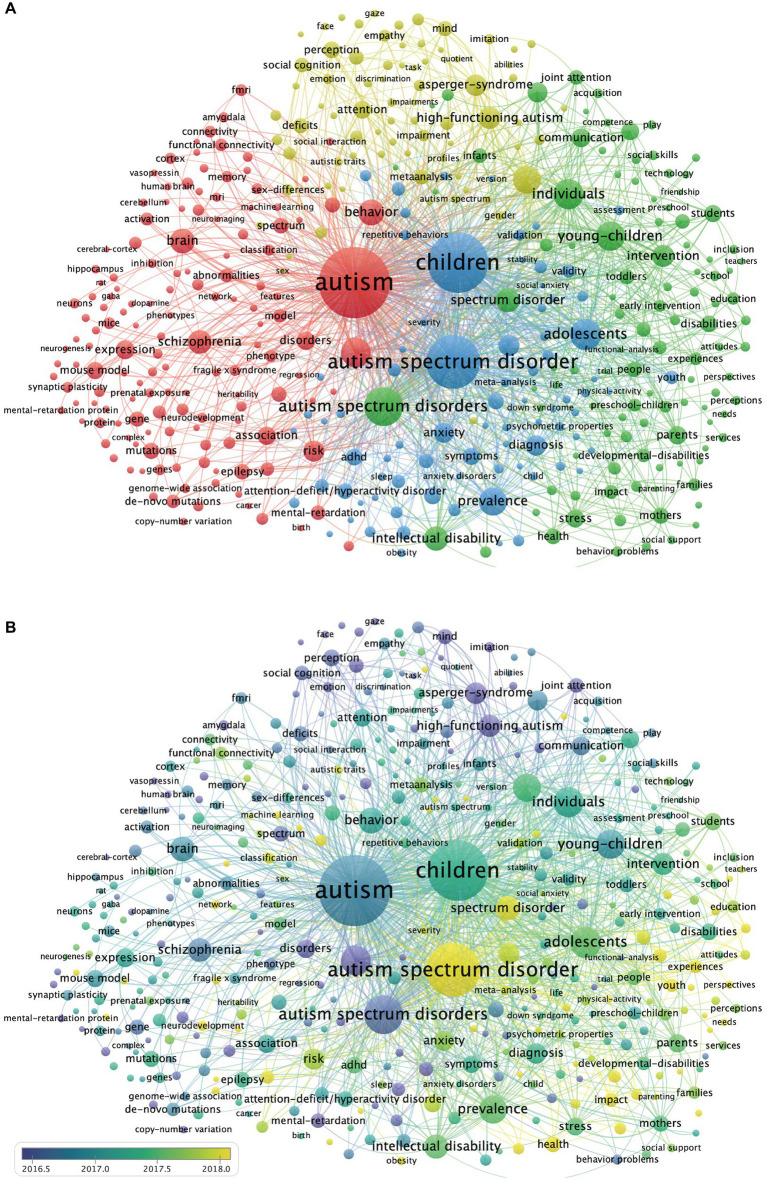
Keywords co-occurrence network. **(A)** Cluster analysis of keywords. There are four clusters of keywords: red indicates Cluster 1 (*n* = 145), green indicates Cluster 2 (*n* = 104), blue indicates Cluster 3 (*n* = 78), yellow indicates Cluster 4 (*n* = 80). **(B)** Evolution of keyword frequency. A minimum number of occurrences of a keyword = 200. Overall, 407 keywords met the threshold criteria. The yellow keywords appear later than purple keywords.

The co-occurrence analysis of keywords in ASD research articles was performed using VOSviewer software; the keywords that occurred ≥200 times were analyzed after being grouped into four clusters of different colors (Figure 6A); the temporal distribution of keywords is summarized in Figure 6B. This map identifies various categories of research: Etiological mechanisms (red), Clinical features (green), Intervention features (blue), and the Asperger cluster (yellow). In the “Etiological mechanisms” cluster, the research includes brain structure and function, genetics, and neuropathology. In the “Clinical features” cluster, the common keywords were “symptoms,” “diagnosis,” “prevalence,” and its comorbidities, including “anxiety” and “sleep.” In the “Intervention features” cluster, the research population of ASD is concentrated in “young children,” “intervention,” and “communication.” These interventions improve the learning and social skills through the involvement of parents and schools.

### The 100 top-cited publications

The screening of the 100 most cited publications on ASD between 2011 and 2022 by Bibliometrix software package, each with >500 citations. The detailed evaluation index information for countries, institutions, journals, and authors (Supplementary Tables S3–S6).

**Figure 7:**
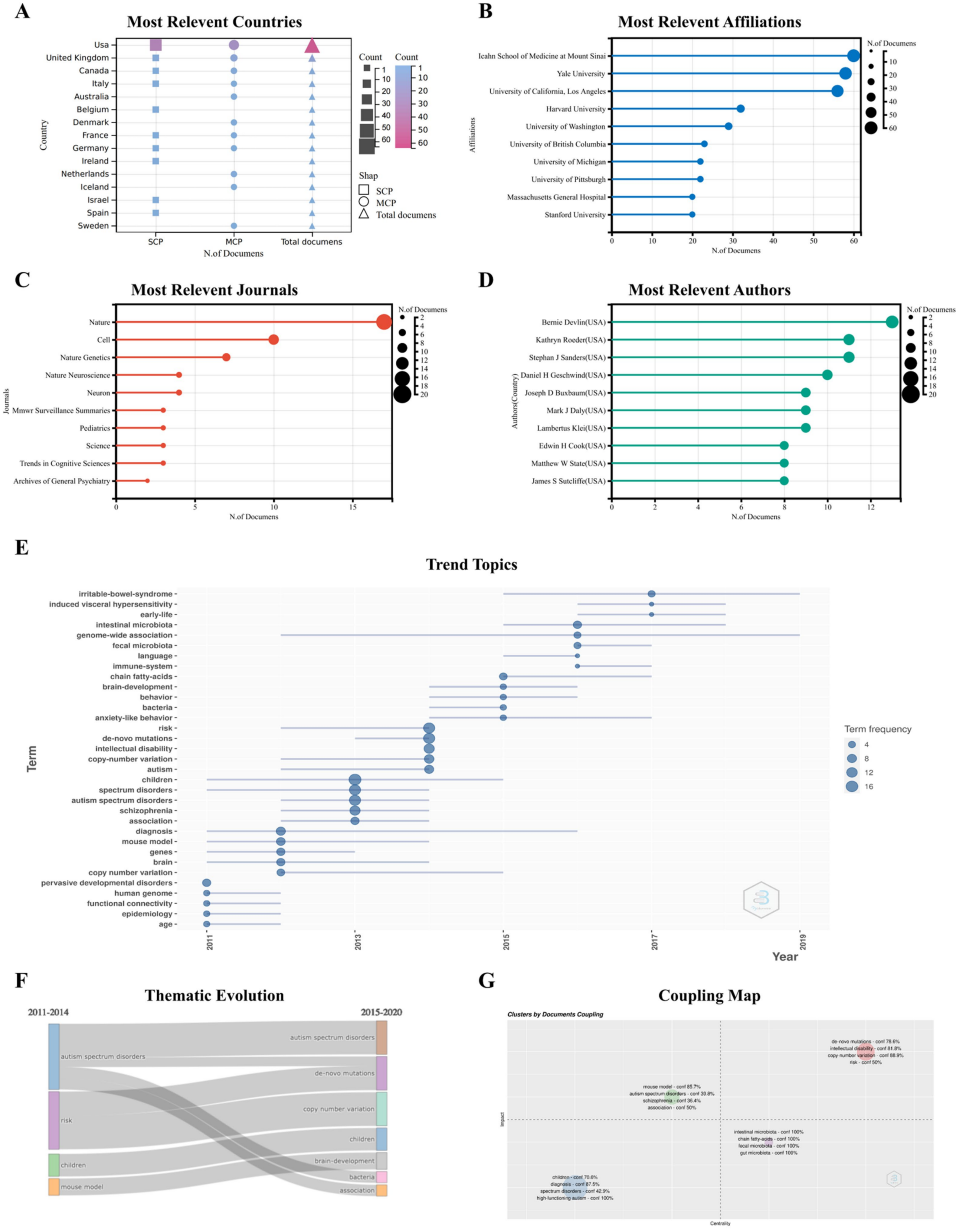
Analysis of the 100 top-cited publications Characteristics of 100 top-cited publications. The most relevant countries **(A)**, affiliations **(B)**, journals **(C)** and authors **(D)**. Trend topics **(E)** and thematic evolution **(F)** of 100 top-cited publication. Coupling Map **(G)**: the coupled analysis of the article, references and keywords is carried out, the centrality of the *x*-axis is displayed, the *y*-axis is the impact, and the confidence (conf%) is calculated.

Taken together, the results indicated that the United States is the country that publishes the most highly cited articles (*n* = 64), including single-country publications (*n* = 37) and multiple-country publications (*n* = 27); most articles are from academic institutions within the USA (Figures 7A,B).

The 100 top-cited ASD publications were published in 48 journals; 17 articles were published in Nature (*n* = 17), making it the highest h-index journal in this list (Supplementary Table S5). In addition, 10 articles were published in Cell, and 7 articles were published in Nature Genetics (Figure 7C). When considering the individual authors’ academic contributions, Bernie Devlin provided 13 publications, followed by Kathryn Roeder and Stephan J Sanders, with 11 publications each (Figure 7D). The details of the top 10 top-cited papers are summarized in Table 5. An article titled “A general framework for estimating the relative pathogenicity of human genetic variants” published by Martin Kircher in Nature Genetics, received the highest number of citations (*n* = 3,353).

**Table 5:** Detail of top 10 citation paper.

Article title	Author/Published year	Journal	IF (2022)	TC
A general framework for estimating the relative pathogenicity of human genetic variants	Kircher et al., 2014	Nature genetics	41.307	3,353
Prevalence of Autism Spectrum Disorder Among Children Aged 8 Years - Autism and Developmental Disabilities Monitoring Network, 11 Sites, United States, 2014	Baio et al., 2018	Morbidity and Mortality Weekly Report	35.301	2,104
Identification of risk loci with shared effects on five major psychiatric disorders: a genome-wide analysis	Smoller et al., 2013	Lancet	202.731	1878
Microbiota modulate behavioral and physiological abnormalities associated with neurodevelopmental disorders	Hsiao et al., 2013	Cell	66.85	1746
Large-scale brain networks and psychopathology: a unifying triple network model	Menon et al., 2011	Trends in cognitive sciences	24.482	1737
Genetic relationship between five psychiatric disorders estimated from genome-wide SNPs	Lee et al., 2013	Nature genetics	41.307	1,449
Synaptic, transcriptional and chromatin genes disrupted in autism	De Rubeis et al., 2014	Nature	69.504	1,436
Sporadic autism exomes reveal a highly interconnected protein network of *de novo* mutations	O’Roak et al., 2012	Nature	69.504	1,426
Neocortical excitation/inhibition balance in information processing and social dysfunction	Yizhar et al., 2011	Nature	69.504	1,405
*De novo* mutations revealed by whole-exome sequencing are strongly associated with autism	Sanders et al., 2012	Nature	69.504	1,329

TC: total citation; IF: impact factor.

The 100 top-cited ASD articles encompassed a range of keywords (Figure 7E) and displayed the main cluster of themes through specific periods (2011–2022) by analyzing those in the selected literature. The Sankey diagrams of thematic evolution explain the topics that evolved throughout the years (Figure 7F). In summary, the core topics of the ASD field in 2011–2014 consisted of the risk of childhood ASD and further developed into the field of human genetic variants, such as CNV and *de novo* mutations. In the subperiod 2015–2020, the further expansion of studies in this field leads to new clusters, such as “immune system,” “brain development,” and “fecal microbiota.” Genome research in the upper right quadrant, including mutations and risk, is a major and evolving theme. The coupled map showing the brain-gut axis field, including intestinal microbiota and chain fatty acids, located in the lower right corner is crucial for autism research but is not yet well-developed (Figure 7G). The research on autism, including animal models, schizophrenia, is a well-developed field, but that on high-functioning autism and diagnosis is a marginal field.

## Discussion

This study used various bibliometric tools and software to analyze the published articles on ASD based on the WoSCC database from 2011 to 2022. By 2022, the annual number of publications and citations of ASD-related research showed an overall upward trend, reflecting the sustained interest and the diversity of areas.

### General information

In terms of regional distribution, researchers from different countries and regions have participated in autism research, and international cooperation has been relatively close over the past decade. The scientific research is supported by several countries and institutions, as well as by large-scale international cooperation (28, 29). The USA has the highest collaboration performance, especially with UK, Canada, Australia and China. In addition to the limitations of financial aid, ethical, cultural, and racial issues are complex constraints that should be overcome for more diversity in autism research (30, 31). We speculated that further collaboration between institutions and countries could promote autism research.

Among the top 20 academic journals, most of the papers were in the Journal of Autism and Developmental Disorders. The frequent publishing of ASD-related papers indicates the interest of readers and journal editors in Autism. Also, substantial studies have been carried out on ASDs, autism, and molecular autism. These journals are ascribed to the field of ASD, focusing on autism research and communication ASD science. However, the analysis of the 10 most cited publications revealed that they were published in such as Nature, Cell, Lancet; these ASD studies were all from high-impact journals.

From the perspective of authors, some of them have made outstanding contributions to global ASD research. Professor Catherine Lord, the top rank for h-index, m-index analysis conducted by the author, and who developed the two gold standards for autism diagnosis (32, 33), are the most influencing factors in the field. ASD is a disease with complex genetic roots. Dr. Catherine Lord has conducted multiple studies using genome-wide association study (GWAS) and gene set analysis to identify variant signatures in autism (34). A recent meta-analysis showed that 74–93% of ASD risk is heritable, with an analysis of CNVs that highlights the key role of rare and *de novo* mutations in the etiology of ASD (35). Variation-affected gene clusters on networks associated with synaptic transmission, neuronal development, and chromatin regulation (36, 37). The identification of the cross-disorder genetic risk factors found by assessing SNP heritability in five psychiatric disorders (38). Five of the top 10 cited papers in Table 5 focus on genetic variation, suggesting that over the past decade, research has shifted from a general concept of genetic risk to the different types of genetic variations associated with autism.

Simon Baron-Cohen of the Autism Research Center at the University of Cambridge was the most published author between 2011 and 2021. He contributed to the mind-blindness hypothesis of autism, developed the autism spectrum quotient (AQ) screening tool for autism, and focused on gender differences in autism (39–41). There are gender/sex differences in the volume and tissue density of brain regions, including the amygdala, hippocampus, and insula, and the heart-blind hypothesis links emotional recognition in individuals with autism to deficits in the amygdala (41–43). Then, Simon et al. backed up the “extreme male brain” theory of autism in a study of 36,000 autistic individuals aged 16–89 (44). Recently, an increasing number of studies from different perspectives have focused on how sex/gender differences are related to autism (4, 5, 45). In the future, studies of neural dimorphism in brain development in autism need to be conducted across the lifespan to reduce age-induced biases (41).

### Hotspots and Frontiers

Keyword analysis was a major indicator for research trends and hotspot analysis. This study shows that keywords for autism research include etiological mechanism, clinical characteristics, and intervention characteristics. Genetic, environmental, epigenetic, brain structure, neuropathological, and immunological factors have contributed to studying its etiological mechanism (46, 47). The studies on the abnormal cortical development in ASD have reported early brain overgrowth (48), reduced resting cerebral blood flow in the medial PFC and anterior cingulate (49), focal disruption of neuronal migration (50), and transcriptomic alterations in the cerebral cortex of autism (51). Genomics studies have identified several variants and genes that increase susceptibility to autism, affecting biological pathways related to chromatin remodeling, regulation of neuronal function, and synaptic development (51–54). In addition, many autism-related genes are enriched in cortical glutamatergic neurons, and mutations in the genes encoding these proteins result in neuronal excitation-inhibitory balance (51, 55). A recent study using single-cell sequencing of the developing human cerebral cortex found strong cell-type-specific enrichment of noncoding mutations in ASD (56). Interestingly, genes interact with the environment; some studies have shown that environmental exposure during pregnancy is a risk factor for brain development (57), and there are changes in DNA methylation in the brains of ASD patients, reflecting an underlying epigenetic dysregulation.

Presently, the diagnosis of ASD is mainly based on symptoms and behaviors, but the disease has a high clinical heterogeneity, and the individual differences between patients are obvious (58). In this study, the keywords of the intervention cluster show the importance of early individualized intervention. Patient data are multidimensional, and individualized diagnoses could be made at multiple levels, such as age, gender, clinical characteristics, and genetic characteristics (59). Early individual genetic diagnosis aids clinical evaluation, ranging from chromosomal microarray (CMA) to fragile X genetic testing (60). However, the results of genetic research cannot guide the treatment. Notably, the treatment of autism is dominated by educational practices and behavioral interventions (61). Medication may address other co-occurring conditions, such as sleep disturbances, epilepsy, and gastrointestinal dysfunction (9). Professor Catherine Lord pointed out that the future of autism requires coordinated, large-scale research to develop affordable, individualized, staged assessments and interventions for people with ASD (62). Professor Baron-Cohen noted that increasing the sample size and collecting data from the same individual multiple times could reduce heterogeneity (58). In addition, screening for objective and valid biomarkers in the future would help to stratify diagnosis and reduce heterogeneity.

According to the keyword trend analysis of 100 highly cited documents, the genetic risk of autism was determined as the hot focus of research, and immune dysregulation and gut microbiome are the new development frontiers after 2015. Patients with ASD have altered immune function, microglia activation was observed in postmortem brain samples, and increased production of inflammatory cytokines and chemokines was observed in cerebrospinal fluid. The microglia are involved in synaptic pruning, and cytokines also affect neuronal migration and axonal projections (63–65). In addition, abnormal peripheral immune responses during pregnancy might affect the developing brain, increasing likelihood of autism (66). Several studies have pointed to abnormalities in immune-related genes in the brain and peripheral blood of autistic patients (51, 67, 68). Immune dysfunction is involved in the etiology of ASD and mediates the accompanying symptoms of autism. The patients have multiple immune-related diseases, asthma, allergic rhinitis, Crohn’s disease, and gastrointestinal dysfunction (69–71). Children with frequent gastrointestinal symptoms, such as abdominal pain, gas, constipation, or diarrhea, had pronounced social withdrawal and stereotyped behavior (70–72). Several studies suggested that these autism-related gastrointestinal problems might be related to intestinal microbiota composition (72–74). Accumulating evidence suggested that the microbiota-gut-brain axis influences human neurodevelopment, a complex system involving immune, metabolic, and vagal pathways in which bacterial metabolites directly affect the brain by disrupting the gut and blood–brain barrier (75–78). Fecal samples from children with autism contained high *Clostridium* species and low *Bifidobacterium* species (79, 80). Probiotics can modulate gut microbiota structure and increase the relative abundance of *Bifidobacteria*, and clinical studies have shown that supplementation with probiotic strains improves attention problems in children with autism (81, 82). Recent clinical trials have shown that microbiota transfer therapy improves gastrointestinal symptoms and autism-like behaviors in children with ASD (83, 84).

## Conclusion

This scientometric study comprehensively analyzes about a decade of global autism research. Research in the field of autism is increasing, with the United States making outstanding contributions, while neuroscience, genetics, brain imaging studies, or studies of the gut microbiome deepen our understanding of the disorder. The study of the brain-gut axis elucidates the mechanism of immunology in autism, and immunological research may be in the renaissance. The current data serve as a valuable resource for studying ASD. However, the future of autism needs further development. In the future, relevant research should be included for a complete representation of the entire autism population, and further collaboration between individuals, institutions, and countries is expected to accelerate the development of autism research.

## Data availability statement

The original contributions presented in the study are included in the article/Supplementary material, further inquiries can be directed to the corresponding authors.

## Author contributions

MJ, DZ, JL, and LW conceived and designed the study. MJ, TL, XL, KY, and LZ contributed to data collection and data analysis. MJ wrote the original manuscript. DZ, JL, and LW revised the article and contributed to the final version of the manuscript. All authors contributed to the article and approved the submitted version.

## Funding

This work was supported by grants from the Key-Area Research and Development Program of Guangdong Province (2019B030335001) and the National Natural Science Foundation of China (grant numbers 82171537, 81971283, 82071541, and 81730037).

## Conflict of interest

The authors declare that the research was conducted in the absence of any commercial or financial relationships that could be construed as a potential conflict of interest.

## Publisher’s note

All claims expressed in this article are solely those of the authors and do not necessarily represent those of their affiliated organizations, or those of the publisher, the editors and the reviewers. Any product that may be evaluated in this article, or claim that may be made by its manufacturer, is not guaranteed or endorsed by the publisher.

## References

[1] Newschaffer CJ Croen LA Daniels J Giarelli E Grether JK Levy SE. The epidemiology of autism spectrum disorders. Annu Rev Public Health. (2007) 28:235–58. doi: 10.1146/annurev.publhealth.28.021406.144007 17367287

[2] Christensen DL Braun KVN Baio J Bilder D Charles J Constantino JN. Prevalence and characteristics of autism spectrum disorder among children aged 8 years - autism and developmental disabilities monitoring network, 11 sites, United States, 2012. MMWR Surveill Summ. (2018) 65:1–23. doi: 10.15585/mmwr.ss6513a1, PMID: 30439868 PMC6237390

[3] Maenner MJ Shaw KA Bakian AV Bilder DA Durkin MS Esler A. Prevalence and characteristics of autism spectrum disorder among children aged 8 years - autism and developmental disabilities monitoring network, 11 sites, United States, 2018. MMWR Surveill Summ. (2021) 70:1–16. doi: 10.15585/mmwr.ss7011a1, PMID: 34855725 PMC8639024

[4] Green RM Travers AM Howe Y McDougle CJ. Women and autism Spectrum disorder: diagnosis and implications for treatment of adolescents and adults. Curr Psychiatry Rep. (2019) 21:22. doi: 10.1007/s11920-019-1006-3, PMID: 30852705

[5] Huang Y Arnold SR Foley KR Trollor JN. Diagnosis of autism in adulthood: a scoping review. Autism. (2020) 24:1311–27. doi: 10.1177/1362361320903128, PMID: 32106698

[6] Loomes R Hull L Mandy WPL. What is the male-to-female ratio in autism spectrum disorder? A systematic review and meta-analysis. J Am Acad Child Adolesc Psychiatry. (2017) 56:466–74. doi: 10.1016/j.jaac.2017.03.013, PMID: 28545751

[7] Geschwind DH. Genetics of autism spectrum disorders. Trends Cogn Sci. (2011) 15:409–16. doi: 10.1016/j.tics.2011.07.003, PMID: 21855394 PMC3691066

[8] Landa R Garrett-Mayer E. Development in infants with autism spectrum disorders: a prospective study. J Child Psychol Psychiatry. (2006) 47:629–38. doi: 10.1111/j.1469-7610.2006.01531.x 16712640

[9] Hyman SL Levy SE Myers SM. Council on children with disabilities SOD, behavioral P. identification, evaluation, and management of children with autism spectrum disorder. Pediatrics. (2020) 145:e20193447. doi: 10.1542/peds.2019-3447 31843864

[10] Lord C Elsabbagh M Baird G Veenstra-Vanderweele J. Autism Spectrum disorder. Lancet. (2018) 392:508–20. doi: 10.1016/S0140-6736(18)31129-2, PMID: 30078460 PMC7398158

[11] Zwaigenbaum L Penner M. Autism spectrum disorder: advances in diagnosis and evaluation. BMJ. (2018) 361:k1674. doi: 10.1136/bmj.k1674 29784657

[12] Bottema-Beutel K Kapp SK Lester JN Sasson NJ Hand BN. Avoiding ableist language: suggestions for autism researchers. Autism Adulthood. (2021) 3:18–29. doi: 10.1089/aut.2020.0014, PMID: 36601265 PMC8992888

[13] Simonoff E Pickles A Charman T Chandler S Loucas T Baird G. Psychiatric disorders in children with autism spectrum disorders: prevalence, comorbidity, and associated factors in a population-derived sample. J Am Acad Child Adolesc Psychiatry. (2008) 47:921–9. doi: 10.1097/CHI.0b013e318179964f, PMID: 18645422

[14] Cooper ID. Bibliometrics basics. J Med Libr Assoc. (2015) 103:217–8. doi: 10.3163/1536-5050.103.4.013, PMID: 26512226 PMC4613387

[15] Chen C Song M. Visualizing a field of research: a methodology of systematic scientometric reviews. PLoS One. (2019) 14:e0223994. doi: 10.1371/journal.pone.0223994, PMID: 31671124 PMC6822756

[16] Carmona-Serrano N Moreno-Guerrero AJ Marin-Marin JA Lopez-Belmonte J. Evolution of the autism literature and the influence of parents: a scientific mapping in web of science. Brain Sci. (2021) 11:74. doi: 10.3390/brainsci11010074, PMID: 33429923 PMC7827242

[17] Rong P Fu Q Zhang X Liu H Zhao S Song X. A bibliometrics analysis and visualization of autism spectrum disorder. Front Psychol. (2022) 13:884600. doi: 10.3389/fpsyt.2022.884600, PMID: 35923445 PMC9339633

[18] Wang K Duan W Duan Y Yu Y Chen X Xu Y. A bibliometric insight of genetic factors in Asd: emerging trends and new developments. Brain Sci. (2020) 11:33. doi: 10.3390/brainsci11010033, PMID: 33396229 PMC7824688

[19] Wang YX Arora R Choi Y Chung HW Egorov VI Frahm J. Implications of web of science journal impact factor for scientific output evaluation in 16 institutions and investigators’ opinion. Quant Imaging Med Surg. (2014) 4:453–61. doi: 10.3978/j.issn.2223-4292.2014.11.16 25525577 PMC4256244

[20] Luo X Duan H He L. A novel riccati equation grey model and its application in forecasting clean energy. Energy. (2020) 205:118085. doi: 10.1016/j.energy.2020.118085, PMID: 32546893 PMC7290234

[21] Yan J Li Y Zhou P. Impact of Covid-19 pandemic on the epidemiology of stds in China: based on the Gm (1,1) model. BMC Infect Dis. (2022) 22:519. doi: 10.1186/s12879-022-07496-y, PMID: 35659579 PMC9166241

[22] Ahmad T. Global research trends in Mers-Cov: a comprehensive bibliometric analysis from 2012 to 2021. Front Public Health. (2022) 10:933333. doi: 10.3389/fpubh.2022.933333, PMID: 35991022 PMC9386292

[23] Hirsch JE. An index to quantify an individual’s scientific research output. Proc Natl Acad Sci U S A. (2005) 102:16569–72. doi: 10.1073/pnas.0507655102 16275915 PMC1283832

[24] Eyre-Walker A Stoletzki N. The assessment of science: the relative merits of post-publication review, the impact factor, and the number of citations. PLoS Biol. (2013) 11:e1001675. doi: 10.1371/journal.pbio.1001675, PMID: 24115908 PMC3792863

[25] van Eck NJ Waltman L. Software survey: Vosviewer, a computer program for bibliometric mapping. Scientometrics. (2010) 84:523–38. doi: 10.1007/s11192-009-0146-3, PMID: 20585380 PMC2883932

[26] Chen C. Searching for intellectual turning points: progressive knowledge domain visualization. Proc Natl Acad Sci U S A. (2004) 101:5303–10. doi: 10.1073/pnas.0307513100, PMID: 14724295 PMC387312

[27] Xu X Feng C. Mapping the knowledge domain of the evolution of emergy theory: a bibliometric approach. Environ Sci Pollut Res Int. (2021) 28:43114–42. doi: 10.1007/s11356-021-14959-3, PMID: 34152539

[28] Flotte TR. The science policy implications of a trump presidency. Hum Gene Ther. (2017) 28:1–2. doi: 10.1089/hum.2016.29037.trf, PMID: 27922749

[29] Gostin LO. Government and science: the unitary executive versus freedom of scientific inquiry. Hast Cent Rep. (2009) 39:11–2. doi: 10.1353/hcr.0.0114, PMID: 19388377

[30] Butrous G. International cooperation to promote advances in medicine. Ann Thorac Med. (2008) 3:79–81. doi: 10.4103/1817-1737.41913, PMID: 19561884 PMC2700441

[31] Watson R. Developing countries need stronger ethical guidelines on research. BMJ. (2007) 334:1076. doi: 10.1136/bmj.39220.615127.DB, PMID: 17525420 PMC1877951

[32] Luyster R Gotham K Guthrie W Coffing M Petrak R Pierce K. The autism diagnostic observation schedule-toddler module: a new module of a standardized diagnostic measure for autism spectrum disorders. J Autism Dev Disord. (2009) 39:1305–20. doi: 10.1007/s10803-009-0746-z, PMID: 19415479 PMC2893552

[33] Zheng S Kaat A Farmer C Kanne S Georgiades S Lord C. Extracting latent subdimensions of social communication: a cross-measure factor analysis. J Am Acad Child Adolesc Psychiatry. (2021) 60:768–782.e6. doi: 10.1016/j.jaac.2020.08.444, PMID: 33027686 PMC8019433

[34] Autism Spectrum Disorders Working Group of The Psychiatric Genomics C. Meta-analysis of Gwas of over 16,000 individuals with autism spectrum disorder highlights a novel locus at 10q24.32 and a significant overlap with schizophrenia. Mol Autism. (2017) 8:21. doi: 10.1186/s13229-017-0137-9, PMID: 28540026 PMC5441062

[35] Tick B Bolton P Happe F Rutter M Rijsdijk F. Heritability of autism spectrum disorders: a meta-analysis of twin studies. J Child Psychol Psychiatry. (2016) 57:585–95. Epub 2015/12/29. doi: 10.1111/jcpp.12499, PMID: 26709141 PMC4996332

[36] Sanders SJ He X Willsey AJ Ercan-Sencicek AG Samocha KE Cicek AE. Insights into autism Spectrum disorder genomic architecture and biology from 71 risk loci. Neuron. (2015) 87:1215–33. doi: 10.1016/j.neuron.2015.09.016, PMID: 26402605 PMC4624267

[37] Pinto D Delaby E Merico D Barbosa M Merikangas A Klei L. Convergence of genes and cellular pathways dysregulated in autism spectrum disorders. Am J Hum Genet. (2014) 94:677–94. doi: 10.1016/j.ajhg.2014.03.018, PMID: 24768552 PMC4067558

[38] Cross-Disorder Group of the Psychiatric Genomics C Lee SH Ripke S Neale BM Faraone SV Purcell SM. Genetic relationship between five psychiatric disorders estimated from genome-wide Snps. Nat Genet. (2013) 45:984–94. doi: 10.1038/ng.2711, PMID: 23933821 PMC3800159

[39] Baron-Cohen S Wheelwright S Skinner R Martin J Clubley E. The autism-spectrum quotient (Aq): evidence from asperger syndrome/high-functioning autism, males and females, scientists and mathematicians. J Autism Dev Disord. (2001) 31:5–17. doi: 10.1023/a:1005653411471, PMID: 11439754

[40] Grove R Baillie A Allison C Baron-Cohen S Hoekstra RA. The latent structure of cognitive and emotional empathy in individuals with autism, first-degree relatives and typical individuals. Mol Autism. (2014) 5:42. doi: 10.1186/2040-2392-5-42, PMID: 25101164 PMC4123248

[41] Lai MC Lerch JP Floris DL Ruigrok AN Pohl A Lombardo MV. Imaging sex/gender and autism in the brain: etiological implications. J Neurosci Res. (2017) 95:380–97. doi: 10.1002/jnr.23948, PMID: 27870420

[42] Ruigrok AN Salimi-Khorshidi G Lai MC Baron-Cohen S Lombardo MV Tait RJ. A meta-analysis of sex differences in human brain structure. Neurosci Biobehav Rev. (2014) 39:34–50. doi: 10.1016/j.neubiorev.2013.12.004, PMID: 24374381 PMC3969295

[43] Baron-Cohen S Ring HA Bullmore ET Wheelwright S Ashwin C Williams SC. The amygdala theory of autism. Neurosci Biobehav Rev. (2000) 24:355–64. doi: 10.1016/s0149-7634(00)00011-7 10781695

[44] Greenberg DM Warrier V Allison C Baron-Cohen S. Testing the empathizing-systemizing theory of sex differences and the extreme male brain theory of autism in half a million people. Proc Natl Acad Sci U S A. (2018) 115:12152–7. doi: 10.1073/pnas.1811032115, PMID: 30420503 PMC6275492

[45] Baron-Cohen S Cassidy S Auyeung B Allison C Achoukhi M Robertson S. Attenuation of typical sex differences in 800 adults with autism Vs. 3,900 controls. PLoS One. (2014) 9:e102251. doi: 10.1371/journal.pone.0102251, PMID: 25029203 PMC4100876

[46] Neuhaus E Beauchaine TP Bernier R. Neurobiological correlates of social functioning in autism. Clin Psychol Rev. (2010) 30:733–48. doi: 10.1016/j.cpr.2010.05.007 20570622

[47] Young AM Chakrabarti B Roberts D Lai MC Suckling J Baron-Cohen S. From molecules to neural morphology: understanding neuroinflammation in autism spectrum condition. Mol Autism. (2016) 7:9. doi: 10.1186/s13229-016-0068-x, PMID: 26793298 PMC4719563

[48] Courchesne E Campbell K Solso S. Brain growth across the life span in autism: age-specific changes in anatomical pathology. Brain Res. (2011) 1380:138–45. doi: 10.1016/j.brainres.2010.09.101, PMID: 20920490 PMC4500507

[49] Ohnishi T Matsuda H Hashimoto T Kunihiro T Nishikawa M Uema T. Abnormal regional cerebral blood flow in childhood autism. Brain. (2000) 123:1838–44. doi: 10.1093/brain/123.9.1838 10960047

[50] Wegiel J Kuchna I Nowicki K Imaki H Wegiel J Marchi E. The neuropathology of autism: defects of neurogenesis and neuronal migration, and dysplastic changes. Acta Neuropathol. (2010) 119:755–70. doi: 10.1007/s00401-010-0655-4, PMID: 20198484 PMC2869041

[51] Voineagu I Wang X Johnston P Lowe JK Tian Y Horvath S. Transcriptomic analysis of autistic brain reveals convergent molecular pathology. Nature. (2011) 474:380–4. doi: 10.1038/nature10110, PMID: 21614001 PMC3607626

[52] Bernier R Golzio C Xiong B Stessman HA Coe BP Penn O. Disruptive Chd8 mutations define a subtype of autism early in development. Cells. (2014) 158:263–76. doi: 10.1016/j.cell.2014.06.017, PMID: 24998929 PMC4136921

[53] Krumm N Turner TN Baker C Vives L Mohajeri K Witherspoon K. Excess of rare, inherited truncating mutations in autism. Nat Genet. (2015) 47:582–8. doi: 10.1038/ng.3303, PMID: 25961944 PMC4449286

[54] Gilman SR Iossifov I Levy D Ronemus M Wigler M Vitkup D. Rare De novo variants associated with autism implicate a large functional network of genes involved in formation and function of synapses. Neuron. (2011) 70:898–907. doi: 10.1016/j.neuron.2011.05.021, PMID: 21658583 PMC3607702

[55] Naaijen J Bralten J Poelmans G consortium I Glennon JC Franke B. Glutamatergic and gabaergic gene sets in attention-deficit/hyperactivity disorder: association to overlapping traits in Adhd and autism. Transl Psychiatry. (2017) 7:e999. doi: 10.1038/tp.2016.273, PMID: 28072412 PMC5545734

[56] Trevino AE Muller F Andersen J Sundaram L Kathiria A Shcherbina A. Chromatin and gene-regulatory dynamics of the developing human cerebral cortex at single-cell resolution. Cells. (2021) 184:5053–5069.e23. doi: 10.1016/j.cell.2021.07.039, PMID: 34390642

[57] Guinchat V Thorsen P Laurent C Cans C Bodeau N Cohen D. Pre-, Peri- and neonatal risk factors for autism. Acta Obstet Gynecol Scand. (2012) 91:287–300. doi: 10.1111/j.1600-0412.2011.01325.x, PMID: 22085436

[58] Lombardo MV Lai MC Baron-Cohen S. Big data approaches to decomposing heterogeneity across the autism spectrum. Mol Psychiatry. (2019) 24:1435–50. doi: 10.1038/s41380-018-0321-0, PMID: 30617272 PMC6754748

[59] Mottron L. A radical change in our autism research strategy is needed: back to prototypes. Autism Res. (2021) 14:2213–20. doi: 10.1002/aur.2494, PMID: 34077611

[60] Jeste SS Geschwind DH. Disentangling the heterogeneity of autism spectrum disorder through genetic findings. Nat Rev Neurol. (2014) 10:74–81. doi: 10.1038/nrneurol.2013.278, PMID: 24468882 PMC4125617

[61] Reichow B Hume K Barton EE Boyd BA. Early intensive behavioral intervention (Eibi) for young children with autism spectrum disorders (Asd). Cochrane Database Syst Rev. (2018) 5:CD009260. doi: 10.1002/14651858.CD009260.pub3, PMID: 29742275 PMC6494600

[62] Lord C Charman T Havdahl A Carbone P Anagnostou E Boyd B. The lancet commission on the future of care and clinical research in autism. Lancet. (2022) 399:271–334. doi: 10.1016/S0140-6736(21)01541-5, PMID: 34883054

[63] Vargas DL Nascimbene C Krishnan C Zimmerman AW Pardo CA. Neuroglial activation and neuroinflammation in the brain of patients with autism. Ann Neurol. (2005) 57:67–81. doi: 10.1002/ana.20315, PMID: 15546155

[64] Bessis A Bechade C Bernard D Roumier A. Microglial control of neuronal death and synaptic properties. Glia. (2007) 55:233–8. doi: 10.1002/glia.20459, PMID: 17106878

[65] Onore C Careaga M Ashwood P. The role of immune dysfunction in the pathophysiology of autism. Brain Behav Immun. (2012) 26:383–92. doi: 10.1016/j.bbi.2011.08.007, PMID: 21906670 PMC3418145

[66] Patterson PH. Immune involvement in schizophrenia and autism: etiology, pathology and animal models. Behav Brain Res. (2009) 204:313–21. doi: 10.1016/j.bbr.2008.12.016, PMID: 19136031

[67] Filosi M Kam-Thong T Essioux L Muglia P Trabetti E Spooren W. Transcriptome signatures from discordant sibling pairs reveal changes in peripheral blood immune cell composition in autism spectrum disorder. Transl Psychiatry. (2020) 10:106. doi: 10.1038/s41398-020-0778-x, PMID: 32291385 PMC7156413

[68] Glatt SJ Tsuang MT Winn M Chandler SD Collins M Lopez L. Blood-based gene expression signatures of infants and toddlers with autism. J Am Acad Child Adolesc Psychiatry. (2012) 51:934–944.e2. doi: 10.1016/j.jaac.2012.07.007, PMID: 22917206 PMC3756503

[69] Kohane IS McMurry A Weber G MacFadden D Rappaport L Kunkel L. The co-morbidity burden of children and Young adults with autism spectrum disorders. PLoS One. (2012) 7:e33224. doi: 10.1371/journal.pone.0033224, PMID: 22511918 PMC3325235

[70] Needham BD Adame MD Serena G Rose DR Preston GM Conrad MC. Plasma and fecal metabolite profiles in autism spectrum disorder. Biol Psychiatry. (2021) 89:451–62. doi: 10.1016/j.biopsych.2020.09.025, PMID: 33342544 PMC7867605

[71] Robinson-Agramonte MLA Noris Garcia E Fraga Guerra J Vega Hurtado Y Antonucci N Semprun-Hernandez N. Immune dysregulation in autism spectrum disorder: what do we know about it? Int J Mol Sci. (2022) 23:3033. doi: 10.3390/ijms23063033, PMID: 35328471 PMC8955336

[72] Chaidez V Hansen RL Hertz-Picciotto I. Gastrointestinal problems in children with autism, developmental delays or typical development. J Autism Dev Disord. (2014) 44:1117–27. doi: 10.1007/s10803-013-1973-x, PMID: 24193577 PMC3981895

[73] Strati F Cavalieri D Albanese D De Felice C Donati C Hayek J. New evidences on the altered gut microbiota in autism spectrum disorders. Microbiome. (2017) 5:24. doi: 10.1186/s40168-017-0242-1, PMID: 28222761 PMC5320696

[74] Hsiao EY McBride SW Hsien S Sharon G Hyde ER McCue T. Microbiota modulate behavioral and physiological abnormalities associated with neurodevelopmental disorders. Cells. (2013) 155:1451–63. doi: 10.1016/j.cell.2013.11.024, PMID: 24315484 PMC3897394

[75] Cryan JF O’Riordan KJ Cowan CSM Sandhu KV Bastiaanssen TFS Boehme M. The microbiota-gut-brain Axis. Physiol Rev. (2019) 99:1877–2013. doi: 10.1152/physrev.00018.2018 31460832

[76] Dinan TG Cryan JF. Gut instincts: microbiota as a key regulator of brain development, ageing and neurodegeneration. J Physiol. (2017) 595:489–503. doi: 10.1113/JP273106, PMID: 27641441 PMC5233671

[77] Fiorentino M Sapone A Senger S Camhi SS Kadzielski SM Buie TM. Blood-brain barrier and intestinal epithelial barrier alterations in autism spectrum disorders. Mol Autism. (2016) 7:49. doi: 10.1186/s13229-016-0110-z, PMID: 27957319 PMC5129651

[78] Srikantha P Mohajeri MH. The possible role of the microbiota-gut-brain-Axis in autism Spectrum disorder. Int J Mol Sci. (2019) 20:2115. doi: 10.3390/ijms20092115, PMID: 31035684 PMC6539237

[79] De Angelis M Piccolo M Vannini L Siragusa S De Giacomo A Serrazzanetti DI. Fecal microbiota and metabolome of children with autism and pervasive developmental disorder not otherwise specified. PLoS One. (2013) 8:e76993. doi: 10.1371/journal.pone.0076993, PMID: 24130822 PMC3793965

[80] Finegold SM Dowd SE Gontcharova V Liu C Henley KE Wolcott RD. Pyrosequencing study of fecal microflora of autistic and control children. Anaerobe. (2010) 16:444–53. doi: 10.1016/j.anaerobe.2010.06.008, PMID: 20603222

[81] Duque A Demarqui FM Santoni MM Zanelli CF Adorno MAT Milenkovic D. Effect of probiotic, prebiotic, and synbiotic on the gut microbiota of autistic children using an in vitro gut microbiome model. Food Res Int. (2021) 149:110657. doi: 10.1016/j.foodres.2021.110657, PMID: 34600659

[82] Grimaldi R Cela D Swann JR Vulevic J Gibson GR Tzortzis G. In vitro fermentation of B-Gos: impact on Faecal bacterial populations and metabolic activity in autistic and non-autistic children. FEMS Microbiol Ecol. (2017) 93:fiw233. doi: 10.1093/femsec/fiw233, PMID: 27856622 PMC5155555

[83] Kang DW Adams JB Gregory AC Borody T Chittick L Fasano A. Microbiota transfer therapy alters gut ecosystem and improves gastrointestinal and autism symptoms: an open-label study. Microbiome. (2017) 5:10. doi: 10.1186/s40168-016-0225-7, PMID: 28122648 PMC5264285

[84] Li N Chen H Cheng Y Xu F Ruan G Ying S. Fecal microbiota transplantation relieves gastrointestinal and autism symptoms by improving the gut microbiota in an open-label study. Front Cell Infect Microbiol. (2021) 11:759435. doi: 10.3389/fcimb.2021.759435, PMID: 34737978 PMC8560686

