# Supplementary material for: Autism spectrum disorder research: knowledge mapping of progress and focus between 2011 and 2022
Source: Front Psychiatry. 2023 Apr 25;14:1096769. doi: 10.3389/fpsyt.2023.1096769 (PMC10168184; doi:10.3389/fpsyt.2023.1096769)
Supplement: Supplementary file 1 [file Table_1.docx]

**Table S1. Main information of All publications**

| **Description** | **Results** |
| --- | --- |
| #1 Main Information About Data | |
| Sources (Journals, Books, etc) | 6346 |
| Documents | 57108 |
| Annual Growth Rate % | 0.16 |
| Average Citations per Document | 21.61 |
| References | 1056125 |
| #2 Document Contents | |
| Keywords Plus (ID) | 38546 |
| Author's Keywords (DE) | 61477 |
| Authors | 133460 |
| #3 Authors Collaboration | |
| Single-authored Documents | 3485 |
| Co-authors per Document | 5.7 |
| #4 Document Types | |
| Article | 46574 |
| Proceedings Paper | 2643 |
| Review | 7891 |

**Table S2. Detail of Top20 co-cited Reference**

| **Rank** | **Reference title** | **Author/Published year** | **Journal** | **IF** | **TC** |
| --- | --- | --- | --- | --- | --- |
| 1 | Diagnostic and Statistical Manual of Mental Disorders, Fifth Edition | American Psychiatric Association,2013 | - | - | 2956 |
| 2 | Prevalence of Autism Spectrum Disorder Among Children Aged 8 Years - Autism and Developmental Disabilities Monitoring Network, 11 Sites, United States, 2014 | Baio et al.,2018 | Morbidity and Mortality Weekly Report | 29.095 | 1789 |
| 3 | Wechsler abbreviated scale of intelligence | David Wechsler,2011 | - | - | 1454 |
| 4 | Global prevalence of autism and other pervasive developmental disorders | Elsabbagh et al.,2012 | Autism research | 4.633 | 1105 |
| 5 | Prevalence of autism spectrum disorder among children aged 8 years - autism and developmental disabilities monitoring network, 11 sites, United States, 2010. | Wingate et al.,2014 | Morbidity and Mortality Weekly Report | 29.095 | 1045 |
| 6 | Synaptic, transcriptional and chromatin genes disrupted in autism | De Rubeis et al.,2014 | Nature | 69.504 | 1024 |
| 7 | Autism | Lai et al.,2014 | Lancet | 202.731 | 999 |
| 8 | Functional impact of global rare copy number variation in autism spectrum disorders | Pinto et al.,2010 | Nature | 69.504 | 938 |
| 9 | Sporadic autism exomes reveal a highly interconnected protein network of de novo mutations | O'Roak et al.,2012 | Nature | 69.504 | 927 |
| 10 | Genetic heritability and shared environmental factors among twin pairs with autism | Hallmayer et al.,2011 | Archives of general psychiatry | - | 924 |
| 11 | The contribution of de novo coding mutations to autism spectrum disorder | Iossifov et al.,2014 | Nature | 69.504 | 899 |
| 12 | De novo mutations revealed by whole-exome sequencing are strongly associated with autism | Sanders et al.,2012 | Nature | 69.504 | 860 |
| 13 | Prevalence and Characteristics of Autism Spectrum Disorder Among Children Aged 8 Years--Autism and Developmental Disabilities Monitoring Network, 11 Sites, United States, 2012 | Christensen et al.,2016 | Morbidity and Mortality Weekly Report | 29.095 | 838 |
| 14 | Prevalence of autism spectrum disorders--Autism and Developmental Disabilities Monitoring Network, 14 sites, United States, 2008 | Baio et al.,2012 | Morbidity and Mortality Weekly Report | 29.095 | 825 |
| 15 | Patterns and rates of exonic de novo mutations in autism spectrum disorders | Neale et al.,2012 | Nature | 69.504 | 757 |
| 16 | De novo gene disruptions in children on the autistic spectrum | Iossifov et al.,2012 | Neuron | 18.688 | 672 |
| 17 | Multiple recurrent de novo CNVs, including duplications of the 7q11.23 Williams syndrome region, are strongly associated with autism | Sanders et al.,2011 | Neuron | 18.688 | 631 |
| 18 | What Is the Male-to-Female Ratio in Autism Spectrum Disorder? A Systematic Review and Meta-Analysis | Loomes et al.,2017 | Journal of the American Academy of Child and Adolescent Psychiatry | 13.113 | 611 |
| 19 | Insights into Autism Spectrum Disorder Genomic Architecture and Biology from 71 Risk Loci | Sanders et al.,2015 | Neuron | 18.688 | 524 |
| 20 | Evidence-Based Practices for Children, Youth, and Young Adults with Autism Spectrum Disorder: A Comprehensive Review | Wong et al.,2015 | Journal of autism and developmental disorders | 4.345 | 509 |

**Table S3. Countries of 100 Top-Cited Articles**

| **Rank** | **Countries** | **ND** | **SCP^a^** | **MCP^b^** | **TC** | **AAC** |
| --- | --- | --- | --- | --- | --- | --- |
| 1 | Usa | 64 | 37 | 27 | 57025 | 891 |
| 2 | United Kingdom | 11 | 3 | 8 | 8535 | 776 |
| 3 | Canada | 4 | 1 | 3 | 3303 | 826 |
| 4 | Italy | 3 | 2 | 1 | 2224 | 741 |
| 5 | Australia | 2 | 0 | 2 | 2483 | 1242 |
| 6 | Belgium | 2 | 2 | 0 | 1294 | 647 |
| 7 | Denmark | 2 | 0 | 2 | 1224 | 612 |
| 8 | France | 2 | 1 | 1 | 1173 | 586 |
| 9 | Germany | 2 | 1 | 1 | 1841 | 920 |
| 10 | Ireland | 2 | 2 | 0 | 1346 | 673 |
| 11 | Netherlands | 2 | 0 | 2 | 1411 | 706 |
| 12 | Iceland | 1 | 0 | 1 | 1194 | 1194 |
| 13 | Israel | 1 | 1 | 0 | 570 | 570 |
| 14 | Spain | 1 | 1 | 0 | 654 | 654 |
| 15 | Sweden | 1 | 0 | 1 | 574 | 574 |

ND: nubmer of documens; TC: total citation; AAC: average article citations

^a^ Articles in which all authors have the same country affiliation are called single country publications (SCP) and are considered to represent intra-country (within) collaboration.

^b^ Articles with authors having different country affiliations are called multiple country publications (MCP) and considered to represent the international collaboration of that country.

**Table S4. Frequent Affiliations of 100 Top-Cited Articles.**

| **Affiliations** | **Country** | **ND** |
| --- | --- | --- |
| Icahn School of Medicine at Mount Sinai | USA | 60 |
| Yale University | USA | 58 |
| University of California, Los Angeles | USA | 56 |
| Harvard University | USA | 32 |
| University of Washington | USA | 29 |
| University of British Columbia | UK | 23 |
| University of Michigan | USA | 22 |
| University of Pittsburgh | USA | 22 |
| Massachusetts General Hospital | USA | 20 |
| Stanford University | USA | 20 |

ND: nubmer of documens

**Table S5. Frequent Journals of 100 Top-Cited Articles**

| **Journals** | **TC** | **ND** | **h_index** | **g_index** | **m_index** |
| --- | --- | --- | --- | --- | --- |
| Nature | 17318 | 17 | 17 | 17 | 1.417 |
| Cell | 7473 | 10 | 10 | 10 | 0.833 |
| Nature Genetics | 8243 | 7 | 7 | 7 | 0.583 |
| Nature Neuroscience | 2823 | 4 | 4 | 4 | 0.333 |
| Neuron | 3069 | 4 | 4 | 4 | 0.333 |
| Mmwr Surveillance Summaries | 4382 | 3 | 3 | 3 | 0.333 |
| Pediatrics | 2469 | 3 | 3 | 3 | 0.250 |
| Science | 2038 | 3 | 3 | 3 | 0.273 |
| Trends in Cognitive Sciences | 3213 | 3 | 3 | 3 | 0.250 |
| Archives of General Psychiatry | 1629 | 2 | 2 | 2 | 0.167 |

TC: total citation; ND: nubmer of documens

**Table S6. Frequent Authors of 100 Top-Cited Articles.**

| **Authors(Country)** | **TC** | **ND** | **h_index** | **g_index** | **m_index** |
| --- | --- | --- | --- | --- | --- |
| Bernie Devlin(USA) | 12236 | 13 | 13 | 13 | 1.083 |
| Kathryn Roeder(USA) | 9780 | 11 | 11 | 11 | 0.917 |
| Stephan J Sanders(USA) | 9858 | 11 | 11 | 11 | 0.917 |
| Daniel H Geschwind(USA) | 8090 | 10 | 10 | 10 | 0.833 |
| Joseph D Buxbaum(USA) | 7635 | 9 | 9 | 9 | 0.818 |
| Mark J Daly(USA) | 9090 | 9 | 9 | 9 | 0.818 |
| Lambertus Klei(USA) | 7289 | 9 | 9 | 9 | 0.750 |
| Edwin H Cook(USA) | 7259 | 8 | 8 | 8 | 0.667 |
| Matthew W State(USA) | 7233 | 8 | 8 | 8 | 0.667 |
| James S Sutcliffe(USA) | 7259 | 8 | 8 | 8 | 0.667 |

TC: total citation; ND: nubmer of documens
